# Partially reduced graphene oxide based FRET on fiber-optic interferometer for biochemical detection

**DOI:** 10.1038/srep23706

**Published:** 2016-03-24

**Authors:** B. C. Yao, Y. Wu, C. B. Yu, J. R. He, Y. J. Rao, Y. Gong, F. Fu, Y. F. Chen, Y. R. Li

**Affiliations:** 1 Key Laboratory of Optical Fiber Sensing and Communications (Education Ministry of China), University of Electronic Science and Technology of China, Chengdu 610054, China; 2 State Key Laboratory of Electronic Thin Films and Integrated Devices, University of Electronic Science and Technology of China, Chengdu 610054, China; 3 Department of Biomedical Engineering, University of Michigan, Ann Arbor, MI 48109, United States; 4 Center for Information in BioMedicine, University of Electronic Science and Technology of China, Chengdu 611731, China

## Abstract

Fluorescent resonance energy transfer (FRET) with naturally exceptional selectivity is a powerful technique and widely used in chemical and biomedical analysis. However, it is still challenging for conventional FRET to perform as a high sensitivity compact sensor. Here we propose a novel ‘*FRET on Fiber*’ concept, in which a partially reduced graphene oxide (prGO) film is deposited on a fiber-optic modal interferometer, acting as both the fluorescent quencher for the FRET and the sensitive cladding for optical phase measurement due to refractive index changes in biochemical detection. The target analytes induced fluorescence recovery with good selectivity and optical phase shift with high sensitivity are measured simultaneously. The functionalized prGO film coated on the fiber-optic interferometer shows high sensitivities for the detections of metal ion, dopamine and single-stranded DNA (ssDNA), with detection limits of 1.2 nM, 1.3 μM and 1 pM, respectively. Such a prGO based *‘FRET on fiber’* configuration, bridging the FRET and the fiber-optic sensing technology, may serve as a platform for the realization of series of integrated ‘*FRET on Fiber*’ sensors for on-line environmental, chemical, and biomedical detection, with excellent compactness, high sensitivity, good selectivity and fast response

Biochemical detection with both high sensitivity and selectivity is of great importance in medical, chemical, environmental and security areas. During the past decades, various optical biochemical detection methods based on diverse technologies have been reported and still developing fast^1^,^2^,^3^,^4^,^5^,^6^,^7^. In particular, the fluorescence resonance energy transfer (FRET) is a highly popular method due to its biochemical universality and naturally exceptional selectivity^8^,^9^,^10^. However, as a key biochemical detection technique, the optical signal of the conventional FRET method based on fluorescent intensity measurement is just collected from dispersions or solutions in free-space. Hence, it is quite challenging to replace the conventional FRET instrument with a compact FRET probe for on-line biochemical detection, limiting the applications of the FRET technology considerably. Optical fiber sensors have great potential to solve such a problem, as they are compact and suitable for on-line applications^11^,^12^,^13^,^14^,^15^. Furthermore, high sensitivity could be achieved with a fiber-optic interferometer based on optical phase measurement due to refractive index changes^16^,^17^. Hence, by combining the FRET and fiber-optic sensing technologies, we could pave a new way for the realization of all-fiber based FRET sensors, which can be expected to have high impact on the area of the biochemical detection due to their excellent compactness, high sensitivity, good selectivity, and fast response.

The emergence of graphene materials provides the possibility to bridge such a combination^18^,^19^, where graphene or graphene oxide acting as a naturally fluorescent quencher can be used to improve the fluorescence transfer efficiency^20^,^21^. Accordingly, a variety of graphene enhanced FRET methods for detection of metal ions, DNAs, and virus have been reported recently^22^,^23^,^24^,^25^. Also, it has been demonstrated that graphene films can be wrapped around or deposited on all-fiber structures to form functional devices^26^,^27^,^28^. Finally, graphene materials are of high surface activity and good molecular adsorption ability, indicating great potential for biochemical detection applications^29^,^30^.

In this Article, by coating a layer of partially reduced graphene oxide film (prGO) on the surface of a fiber-optic modal interferometer, we construct a prGO-based *‘FRET on Fiber’* platform for biochemical detection. In this *‘FRET on Fiber’* approach, we use Rhodamine 6G (Rh6G) as the fluorescent donor, while the prGO film works as the acceptor, the molecular binder and the evanescent field enhancer. Hence, the binding competition induced FRET and all-fiber optical interference occur simultaneously on the fiber surface where the prGO film acts as the sensing element. Good selectivity and high sensitivity can be achieved by measuring the fluorescent recovering in the visible range and the interferometric phase change in telecommunication wavelength range, simultaneously. Taking full advantage of the high resolution of the wavelength detection from the optical modal interference (1 pm), the detection limits for Cadmium ion (1.2 nM), dopamine (1.3 μM) and ssDNA (1 pM) are achieved, respectively.

## Results

### Concept of the *‘FRET on Fiber’* approach

**Figure 1 f1:**
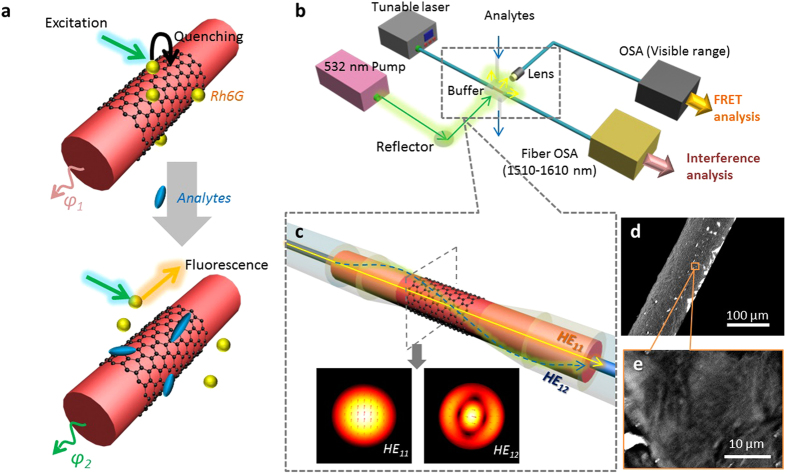
Concept and structure. (**a**), shows the principle of the graphene based *‘FRET on fiber’*. Due to the competition on the prGO, fluorescent restoration and optical phase modulation can occur simultaneously. (**b**), schematically demonstrates the detection system containing two channels. One is for fluorescence detection, and the other is for all-fiber interference phase measurement. The prGO coated fiber-optic modal interferometer is integrated in the detection system and fixed in a buffer with effective volume of 0.1 mL. Analytes are injected in and removed from the buffer. (**c**), illustrates the probe structure in details. prGO film (black hexagons) is deposited around the etched MMF section. In the interferometer, interference between HE_11_ and HE_12_ mode occurs, while the HE_12_ mode is enhanced in the prGO-coated area (simulated by FEM method, *COMSOL*). (**d**), the scanning electronic microscope (SEM) image, in which the dark film is the prGO film. (**e**), the zoom-in of (**d**).

The concept of the prGO based *‘FRET on fiber’* detection configuration is schematically shown in Fig. 1a. prGO is coated around an optical fiber, and Rh6G molecules are adsorbed on the surface of the prGO. Due to the quenching effect of the prGO, originally there is almost no fluorescent scattering from the Rh6G. Once a specific analyte is injected into the sampling area, due to the binding competition, Rh6G molecules are released, and the fluorescent intensity restoration could be observed^31^,^32^. Simultaneously, during this process, the effective refractive index of the prGO-coated area varies as a function of the analyte concentration. By taking advantage of optical modal interference, the phase difference could be accurately measured^33^.

Accordingly, we built an experimental setup for biochemical detection, in which a prGO-coated fiber-optic modal interferometer is designed as the sensor. In Fig. 1b, a tunable laser over a range of 1510–1590 nm (CW, average power of 10 mW) is launched into the prGO-coated fiber-optic modal interferometer, and the output interference spectrum is collected and analyzed by an optical spectrum analyzer (OSA), with wavelength resolution of up to 0.1 pm. In order to excite the fluorescence, a 532 nm pulsed laser (pulse width 5 ns, peak power 2.2 W) is focused onto the prGO–coated area, here the fluorescent signal is collected by a lens and measured by the spectroscopy. In the sample chamber, the interferometer is tightly fixed in a microfluidic buffer (Supplementary Fig. S1). Through the experiment, the volume of all the solution samples is fixed 60 μL. The prGO-coated fiber-optic modal interferometer is composed by a section of multimode fiber sandwiched between two single mode fibers, known as the singlemode-multimode-singlemode (SMS) structure coated with a layer of prGO film, as shown in Fig. 1c 34,35. In this work, the multimode fiber length is ~3.2 cm, to form only one interference dip in the wavelength range of 1510 nm to 1590 nm. The prGO film is deposited onto the etched multimode fiber section with a cladding diameter of ~90 μm. The coated prGO has a length of ~3 cm and a thickness of ~2 μm. In conventional FRET methods, GO quencher is dispersed in the free solution, while in this work the prGO works both as a quencher and sensitive film tightly wrapped around the fiber (See fabrication process in Supplementary Fig. S2). The optical interference between the HE_11_ and HE_12_ modes produces the resonant dip in the spectra. By using the finite element method in software *COMSOL*, we simulate the comparison of the electric field distributions of HE_11_ and HE_12_ modes in the etched multimode fiber and in the prGO-coated multimode fiber. Due to the prGO induced refractive index modulation, the HE_12_ mode evanescent field could be enhanced, which is helpful for improving the detection sensitivity^36^ (Supplementary Figs S4 and S7). Figure 1d[Fig f1]e provide the scanning electron microscope (SEM) picture of the prGO-coated fiber section. Compared with the monolayer graphene grown by the CVD method, the prGO reduced from GO is rougher and darker. (Supplementary Information S3).

### The prGO film

To realize the *‘FRET on Fiber’* detection, the prGO was fabricated by reducing GO in the hot Vitamin C for 20 minutes^37^,^38^. (Supplementary Information S2) GO could either emit fluorescence or quench fluorescence, due to its heterogeneous chemical, atomic and electronic structures of GO^21^. When GO performs as the fluorescent quencher, its sp2 domains determine its quenching efficiency, which could be further improved after reduction^39^,^40^. Conventionally, GO as an ideal fluorescence quencher with good binding competition ability is widely used in FRET methods. However, as the GO is hydrophilic and easily dissolved in water, it is not suitable to use it as a film to detect the concentration of aqueous analytes. The prGO film (or graphene film), is hydrophobic, it can keep stable as a film on fiber in the aqueous environment. However, due to the rGO film (or graphene film) usually contains few functional group, which limited its potential application in selective and self-reference biochemical sensing. In order to address such issues, here we designed and prepared a prGO material to instead of the conventional rGO or GO to form a multi-functional film. It can be deposited onto the fiber as a reliable film to detect the ion or molecular contraction in the free solution. This prGO film contains sufficient functional groups to bind molecules, and could improve the FRET efficiency and sensitivity, hence, it performs as a fluorescent acceptor, a molecular adsorber, and also an optical evanescent field enhancer on the fiber-optic modal interferometer in this structure.

**Figure 2:**
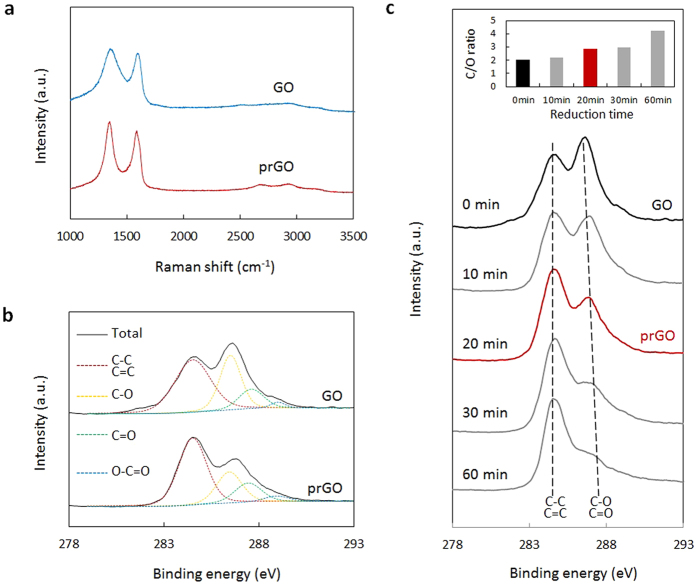
Characterization of the prGO film. (**a**), Raman scattering spectra of the GO (blue curve) and the prGO (red curve), high D peak and G peak indicate that the prGO film is of μm level thickness. (**b**), XPS curves of the GO and prGO. Carbon atoms in: C-C/C = C key (red, with peak at 284.6 eV), C-O key (yellow, with peak at 286.5 eV), C = O key (green, peak at 287.6 eV), O-C = O key (blue, peak at 289 eV). (**c**), XPS curves of rGO samples, reduced from GO (black curve) from 0 ~ 60 min. The prGO film in this work is moderately reduced (red curve). Inset: C/O ratio of the rGO samples.

Figure 2a shows the Raman spectra of both the prGO and the GO. The prGO keeps the properties of the GO, while the D-peak of the prGO is narrower than that of the GO. It verifies that the prGO has less defects, which is helpful for coating it on a fiber densely. Figure 2b compares the X-ray photoelectron spectra (XPS) of the GO and the prGO. After 20 minute reduction, the C-O, C = O, and O-C = O bonding peaks decrease dramatically, but still exist. That means, there are still lots of groups containing O atoms remained in the prGO. It is noted that the C/O ratio of prGO (after reduced by 20 minutes) is 2.84, much higher that of GO (2.07) before reduction. Figure 2c lists the XPS spectra of the rGO samples reduced from the GO, with reduction time varying from 0 min to 60 min. From the inset of Fig. 2c, one can observe that the ratio of C:O gradually increases from 2.07 to 3.90 with increasing the reduction time from 0 to 60 minutes. Such a prGO could not only be deposited on fiber as a reliable film instead of dispersive in water, but also provide some oxygenated groups to support the binding competitions.

### The prGO-coated fiber-optic modal interferometer

**Figure 3:**
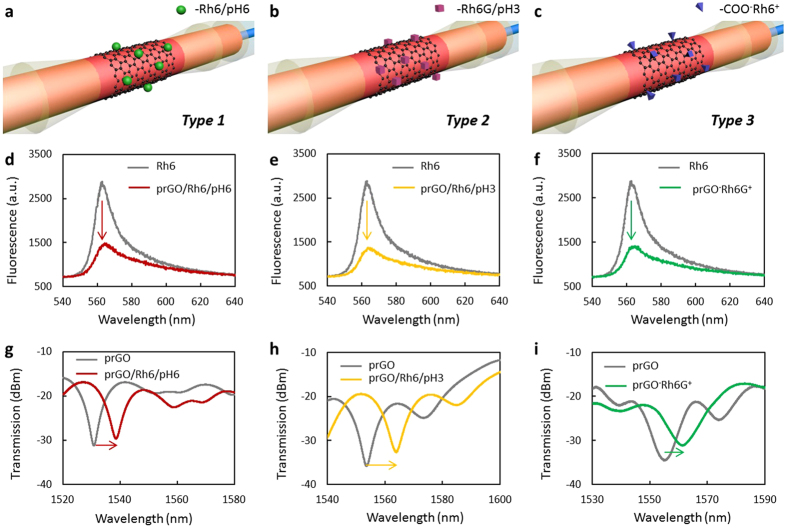
Functionalization of the probes. (**a–c**), shows the functionalized *Type 1, Type 2, Type 3,* schematically. (**d–f**), fluorescence quenched by the prGO film of the *Type 1* (red curve, d), *Type 2* (yellow curve, e) and *Type 3* (green curve, f). Before quenched by the prGO, the fluorescence of the Rh6G is shown as grey curves. (**g–i**), show the optical spectra of the *Type 1* with dip location of 1538.2 nm (red curve, g), *Type 2* with dip location of 1563.7 nm (yellow curve, h), *Type 3* with dip location of 1561.1 nm (green curve, i), respectively. Here the grey curves show the spectra before adding Rh6G.

In order to detect Cd^2+^, DA and ssDNA respectively, we functionalized the prGO coated interferometer to be three types. For Cd^2+^ ions, the prGO coated interferometer is used directly (*Type 1*)^41^,^42^. Specifically, to make the *Type 2* not absorbable to ions, and immune to DA agglomeration, they are pre-immersed in nitrate (*Type 2*)^43^,^44^,^45^. For the ssDNA detection, the prGO coated interferometers are pre-functionalized via Na^+^, to form COO^−^Na^+^ bindings (*Type 3*)^46^ (Supplementary Fig. S8). Figure 3a–c show the structures of the three types schematically. Then, 60 μL Rh6G aqueous solution (300 μM) is injected in the sample buffer respectively (Keeping the pH of the buffer for *Type 1, Type 2, Type 3* to be 3.0, 6.0, 7.0, respectively). Figure 3d–f shows the fluorescence of Rh6G before (~2800 a.u.) and after (<1400 a.u.) quenched by the prGO. As the prGO is coated on the surface of the etched fiber rather than dispersed in solution, the fluorescent quenching efficiency is limited by the interaction area. Figure 3g–i illustrates the spectra of the three types of probes before and after immersed in Rh6G. Here, the interference dip shifts are related to both the surrounding refractive index change and the Rh6G adsorption.

### Fluorescent restoration on fiber

**Figure 4:**
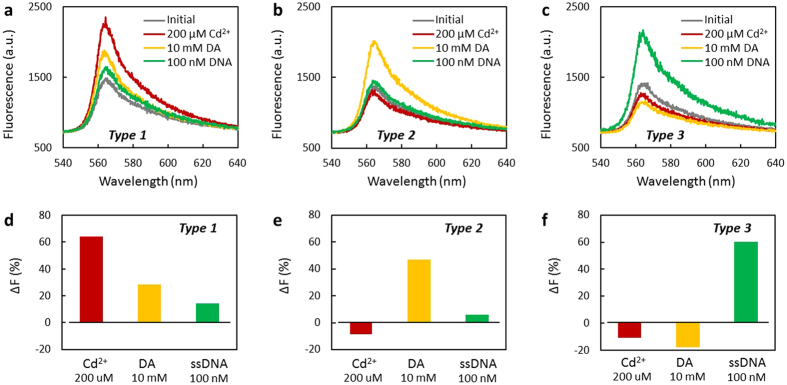
FRET based selectivity. (**a**), Fluorescent spectra of the *Type 1*, when immersed in 200 μM Cd^2+^ (red curve), 10 mM DA (yellow curve), 100 nM DNA (green curve). (**b**), Fluorescent spectra of the *Type 2*, when immersed in 200 μM Cd^2+^ (red curve), 10 mM DA (yellow curve), 100 nM DNA (green curve). (**c**), Fluorescent spectra of the *Type 3*, when immersed in 200 μM Cd^2+^ (red curve), 10 mM DA (yellow curve), 100 nM DNA (green curve). (**d–f**), Histograms: the fluorescent restoration ratio of *Type 1* (**d**), *Type 2* (**e**) and *Type 3* (**f**), respectively.

Taking advantage of the specific binding competitions, the fluorescent restoration of the *Type 1, Type 2, and Type 3* shows dramatic selectivity. The fluorescent responses of the *Type 1, Type 2* and *Type 3* for different analytes are shown in Fig. 4a–c, respectively. Firstly, we pre-tested the initial fluorescent spectra of them immersed in 300 μM Rh6G, as the grey curves shown (corresponding to the Fig. 3d–f). Then, we prepared the solution samples of Cd^2+^ (200 μM, composed by 30 μL/600 μM Rh6G and 30 μL/400 μM Cd^2+^), DA (10 mM, composed of 30 μL/600 μM Rh6G and 30 μL/20 mM DA), and ssDNA (100 nM, composed by 30 μL/600 μM Rh6G and 30 μL/200 nM ssDNA). During the experiment, all the samples were kept stable, all the devices were fixed, and the focus of the pump laser on the samples was carefully kept the same, ensuring the measurements comparable and repeatable.

For Cd^2+^, *Type 1* shows the highest fluorescent restoration (from 1400 a.u. to 2260 a.u.), due to the Cd^2+^-prGO binding takes place of the Rh6G-prGO binding. Oppositely, affected by Cd^2+^, the fluorescent intensities of both *Type 2 and Type 3* decreased. For DA detection, the fluorescent recovery of *Type 2* is mostly obvious (from 1370 a.u. to 1990 a.u.). In comparison, as the pH of *Type 1* is higher and *Type 3* was pre-functionalized by Na^+^, the changes in the fluorescent intensities of both *Type 1* and *Type 3* are much less. Finally, *Type 3* verifies it can recognize ssDNA best, with fluorescence recovering from 1390 a.u. to 2070 a.u. in the sDNA solution, while no obvious changes of the fluorescent intensities for *Type 1* and *Type 2* were observed.

Accordingly, by defining the fluorescent restoration ratio Δ*F* = *(F*_*R*_ − *F*_*Q*_*)/(F*_*I*_ − *F*_*Q*_), Fig. 4d–f concludes the selectivity for the three types. Here *F*_*R*_ is the intensity of recovered fluorescence, *F*_*Q*_ is the intensity of fluorescence quenched by the prGO on fiber, *F*_*I*_ is the initial fluorescence of the 300 mM Rh6G. For *Type 1*, the Δ*F* of Cd^2+^ is estimated to be 64.3%, while its Δ*F* for either DA or ssDNA is lower than 28%. For *Type 2*, the Δ*F* of DA is estimated to be 47.1%, while the Δ*F* for the other two analytes is ignorable. For *Type 3*, the Δ*F* of ssDNA is as high as 60.2%, while the Δ*F* of DA or Cd^2+^ is negative. The experimental results illustrates that the existence of the Cd^2+^, DA and ssDNA could be dramatically visualized by the three types respectively, via FRET based competitions, which enables the *Type 1, Type 2* and *Type 3* being selective Cd^2+^, DA and ssDNA detector, respectively. Moreover, to investigate the Cd^2+^, DA and ssDNA from a hybrid resolution, one can use the three types simultaneously and analyze the results synthetically.

### Sensitivity

**Figure 5:**
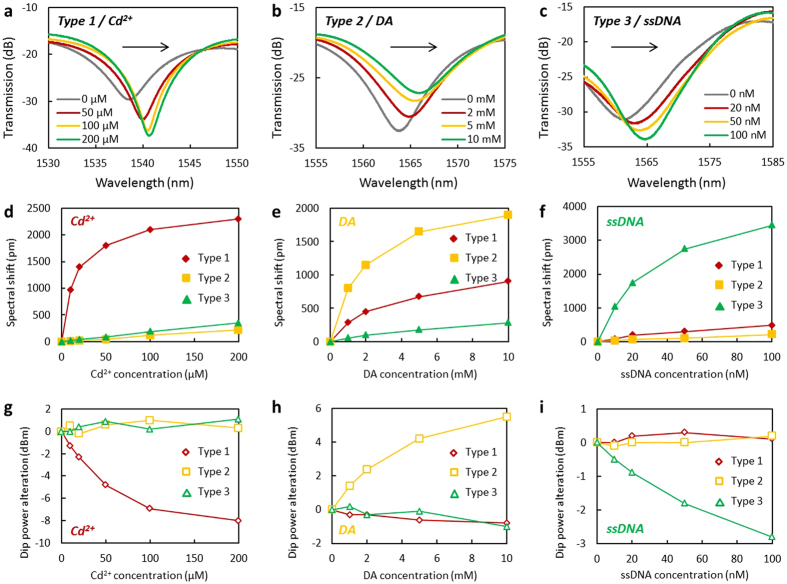
Interference based sensitivity. (**a**), Spectra of the *Type 1* in Cd^2+^ detection (grey to green: 0 μM, 50 μM, 100 μM and 200 μM). (**b**), Spectra of the *Type 2* in DA detection (grey to green: 0 mM, 2 mM, 5 mM and 10 mM). (**c**), Spectra of the *Type 3* in ssDNA detection (grey to green: 0 nM, 20 nM, 50 nM and 100 nM). (**d–f**), for Cd^2+^, DA and ssDNA, the correlation of concentration and spectral shift (red diamonds: *Type 1*, yellow boxes: *Type 2*, green triangles: *Type 3*). (**g–i**), for Cd^2+^, DA and ssDNA, the correlation of concentration and dip power alteration (red diamonds: *Type 1*, yellow boxes: *Type 2*, green triangles: *Type 3*). Here all the spectral shifts and dip alterations are plotted from 0.

Once recognized by the specific types, due to the molecular adsorption energy and charge transfer, the effective index of prGO was changed, so the concentration of the Cd^2+^, DA and ssDNA can be accurately measured by the channel of the optical interference simultaneously. Figure 5a shows the spectra of the *Type 1* for Cd^2+^ detection. When the concentration of Cd^2+^ varies from 0 μM to 200 μM, the interference dip location shifted from 1538.2 nm to 1540.5 nm. Figure 5b shows the spectral shift of the *Type 2* for DA detection. When the concentration of DA increased from 0 mM to 10 mM, the interference dip shifted from 1563.7 nm to 1565.6 nm. Figure 5c shows the spectra of the *Type 3* in ssDNA solution. When the concentration of ssDNA is adjusted from 0 nM to 100 nM, the interference dip location of the *Type 3* changed from 1561.1 nm to 1564.55 nm. In the measurements, the volume of the solutions and the content of the Rh6G of all the solutions were the same.

Figure 5d–f indicate the spectral shifts of the three types of probes, for Cd^2+^, DA, ssDNA detection, respectively. Firstly, for Cd^2+^ detection, the *Type 1* shows the best sensitivity, when the concentration of Cd^2+^ is <10 μM. *Type 1* shows a sensitivity higher than 90 pm/μM, while the other two types are of low response. Secondly, for DA detection, the *Type 2* shows the best sensitivity. Meanwhile, *Type 1* also shows good sensitivity to DA. For ssDNA detection, when the concentration of ssDNA is <10 nM, the *Type 3* shows a sensitivity of ~100 pm/nM. Considering the resolution of the OSA is 0.1 pm, the detection limit of *Type 1, Type 2, Type 3* for Cd^2+^, DA and ssDNA detection is estimated to be 1.1 nM, 1.3 μM and 1 pM, respectively. We also note that, the curve of the wavelength shift versus concentration of Cd^2+^/*Type 1,* DA/ *Type 2,* and ssDNA/ *Type 3* are nonlinear, due to the sensing principle that the sensors detect the molecular adsorption as well as the refractive index changes. Hence, during the spectra shift, when the adsorptions tend to be saturated, the sensitivities decrease. This phenomenon can also be verified by measuring the ‘concentration-dip power’ correlations, as shown in Fig. 5g–i. For Cd^2+^, DA, ssDNA detection, the depth of the wavelength dips of *Type 1, Type 2*, and *Type 3* gradually decreased. In comparison, if the spectral shift is induced just by the liquid index alteration in the buffer, the dip power almost keeps no change (Supplementary Fig. S7). In addition, we measured the response time and the repeatability of the types, detailed in Supplementary Fig. S9. For conventional FRET methods, common response time is in minutes, while the ‘*FRET on Fiber*’ types respond in seconds, which makes them possible for real-time on-line applications.

## Discussions

In the past decade, based on the fiber-optic sensing technology, the concept of *‘Lab on Fiber’* for biochemical detection has been investigated extensively, due to its exceptional practicability^47^. However, enabling biochemical selectivity of fiber-optic sensors still remains as a big challenge. In recent years, based on the measurement of refractive index changes, various all-fiber optic sensors were reported to detect biochemical analytes, such as fiber Bragg gratings and long-period fiber gratings. However, most of them are lack of selectivity, while to modify them by using biochemical materials (such as enzymes, probe DNAs, or antigens) is of high cost and complexity^48^,^49^,^50^. On the other hand, as a powerful means for biochemical analysis, FRET is commonly operated in free-space aqueous environment, as an instrument. So, it would be ideal if one can realize an integrated FRET probe with compact size rather than a bulk instrument. By depositing the prGO film on the surface of optical fiber, we successfully demonstrated that such a graphene based *‘FRET on Fiber’* probe, which not only shows good chemical selectivity due to the FRET mechanism, but also inherits the merits of fiber-optic modal interferometers, i.e. high sensitivity. When compared with conventional FRET methods, the *‘FRET on Fiber’* approach offers much higher sensitivities, as shown in Table 1. Here, the detection limits of this work are estimated based on the maximum resolution of the OSA (0.1 pm).

Moreover, the prGO based fiber-optic FRET sensor could be promoted and optimized by further diversifying FRET materials, fiber-optic interferometer structures, and fluorescent donors. It is expected that a variety of graphene based *‘FRET on Fiber’* probes with different biochemical detection features could be realized in the future.

In conclusion, by combining the prGO based FRET with fiber-optic interferometric sensing, we have successfully demonstrated a *‘FRET on Fiber’* structure for biochemical detection, in which the graphene oxide is partially reduced by using vitamin C to form a reliable film on the surface of optical fibers. The advantages of using the partially reduced graphene oxide film are threefold, that is, it acts as molecular adsorber, fluorescent acceptor/quencher, and evanescent field enhancer simultaneously. By taking advantages of both FRET and optical fiber-optic interferometric sensing synthetically, such a prGO based *‘FRET on Fiber’* sensor has series of significant merits, such as excellent compactness, good selectivity, high sensitivity, and fast response. The obtained limit of detection for cadmium ion, dopamine and ssDNA all reaches single nM, μM and pM level. This work may build a new platform for the realization of series of *‘FRET on Fiber’* sensors for on-line environmental, chemical, and biomedical detection applications.

## Methods

### Experimental arrangement

A Tunable laser (81960A, Agilent, USA, average power 10 mW, range 1510–1590 nm), the prGO-coated fiber-optic modal interferometer, and OSA-1 (8163B, Agilent, USA, resolution 0.1 pm) are connected by SMF to form the interference channel. Beam from a pulsed pump laser (Surelit I, Continuum, USA, peak power 2.2 W), is used to excite fluorescence, and the excited fluorescence is detected by OSA-2 (SR-500iC, Andor, EU), via a lens. (Supplementary Fig. S1).

### Fabrication of the prGO-coated interferometer

A fiber-optic modal interferometer was fabricated by fusion splicing a section of multimode fiber (MMF, core diameter 105 μm, Corning) in between two single mode fibers (SMF-28e, Corning) , with a multimode cavity length of ~3.2 cm. Then, the silica cladding was etched off by Hydrofluoric Acider until a 90 μm cladding in diameter was achieved. GO was fabricated by Hummers’ method, similar to our previous report^55^,^56^ and dispersed in aqueous solution. Then the etched fiber-optic modal interferometer fixed on a substrate was immersed in the GO dispersion. Finally, the GO was partially reduced by Vitamin C aqueous solution (30 g/L) under 80 ^°^C for 20 min^57^. (Supplementary Fig. S2) The prGO based interferometer was finally dried up and characterized by optical microscope, scanning electronic microscope, and Raman spectrometer. (Supplementary Fig. S3).

### Functionalization of the samples and fluorescent measurement

To form *Type 1*, the interferometer samples are directly immersed in Rh6G. **b,** To form Sensor 2, the buffer and the samples are kept in pH ~3 by using HNO_3_ during the measurement, which can prevent the binding of Cd^2+^-prGO. **c,** To form *Type 2*, firstly, the samples are immersed in 5% Na_2_CO_3_ solution for 10 min, then are cleaned by using enough distilled water to wash away the residual Na^+^. (Supplementary Fig. S6).

### Binding competition principles

*Chemical equations of Sensor 1, Sensor 2, and Sensor 3 are shown in Supplementry* Eq. 1 *to* Eq. 7*, respectively.*


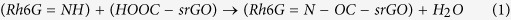



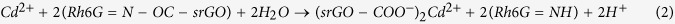



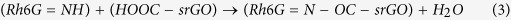



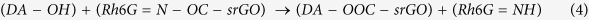



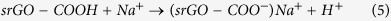



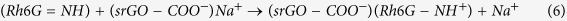



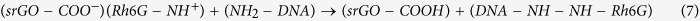


## Additional Information

**How to cite this article**: Yao, B. C. *et al*. Partially reduced graphene oxide based FRET on fiber-optic interferometer for biochemical detection. *Sci. Rep.* **6**, 23706; doi: 10.1038/srep23706 (2016).

## Supplementary Material

Supplementary Information

## Figures and Tables

**Table 1:** Estimated detection limits for FRET in water and on fiber.

	**This work**	**Conventional FRET detection method**
Cd^2+^	1.1 nM (Type 1)	0.5 μM 41, 2~6 μM^51^, 2.38 μM^52^, 0.27 μM^53^
DA	1.3 μM (Type 2)	~2 mM^42^, ~0.1 mM^54^
ssDNA	1 pM (Type 3)	0.01 nM^46^, ~0.1 nM^51^
