# Supplementary material for: Partially reduced graphene oxide based FRET on fiber-optic interferometer for biochemical detection
Source: Sci Rep. 2016 Mar 24;6:23706. doi: 10.1038/srep23706 (PMC4806316; doi:10.1038/srep23706)
Supplement: Supplementary Information [file srep23706-s1.pdf]

Supplementary Information

Partially reduced graphene oxide based FRET on fiber-optic interferometer for biochemical detection

B. C. Yao[1], Y. Wu[1,4]*, C. B. Yu[1], J. R. He[2], Y. J. Rao[1]*, Y. Gong[1,3,4], F. Fu[2] , Y. F. Chen[2] and Y. R. Li[2]

[1]*Key Laboratory of Optical Fiber Sensing and Communications (Education Ministry of China), University of Electronic Science and Technology of China, Chengdu 610054, China*

[2]*State Key Laboratory of Electronic Thin Films and Integrated Devices, University of Electronic Science and Technology of China, Chengdu 610054, China*

[3]*Department of Biomedical Engineering, University of Michigan, Ann Arbor, MI 48109, United States*

[4]*Center for Information in BioMedicine, University of Electronic Science and Technology of China, Chengdu 611731, China*

Corresponding Author: *wuyu@uestc.edu.cn*, *yjrao@uestc.edu.cn*

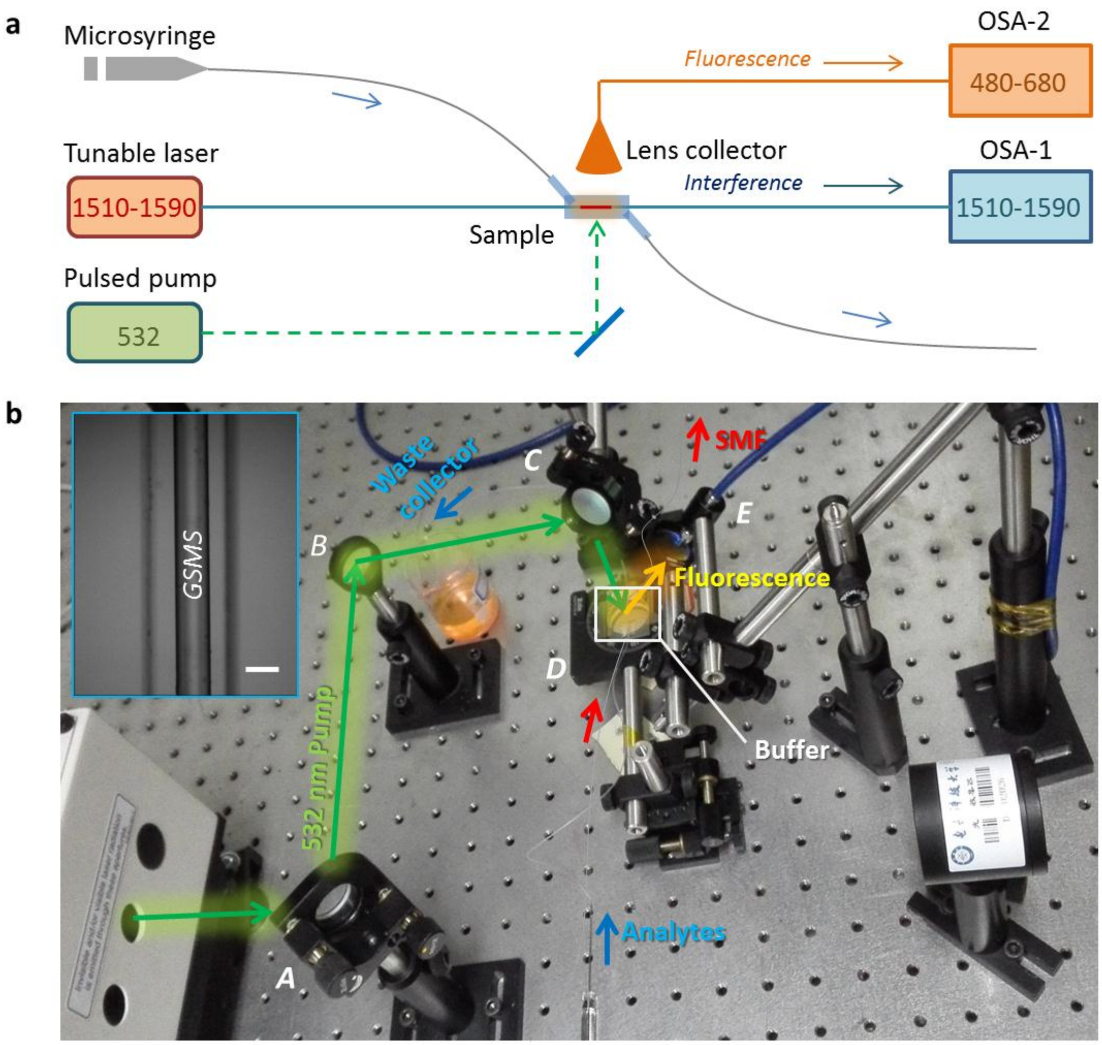

Fig. S1 | Experimental arrangement.

a, Setup and apparatus: Tunable laser (81960A, Agilent, USA, average power 10 mW, range 1510 nm – 1590 nm), Pulsed pump (Surelit I, Continuum, USA, peak power 2.2 W), Telecom optical spectral analyzer (OSA-1, 8163B, Agilent, USA, sampling rate 0.2 pm), OSA-2 (SR-500iC, Andor, EU). **b,** Light path: The pulsed laser is guided by reflector *A*, *B*, *C* and then excites the fluorescence of the prGO based SMS (GSMS) located on stage *D*. The fluorescence is collected by a fiber end via a lens *E* finally. In this picture, SMF sections are marked by red arrows, and analytes are injected and collected via a micropipe marked by blue arrows. The buffer is in the white box. **Inset:** Microscope image of the GSMS in the buffer. The interferometric section packaged in the buffer is fixed straightly to avoid bending. Here the bar is 100 μm.

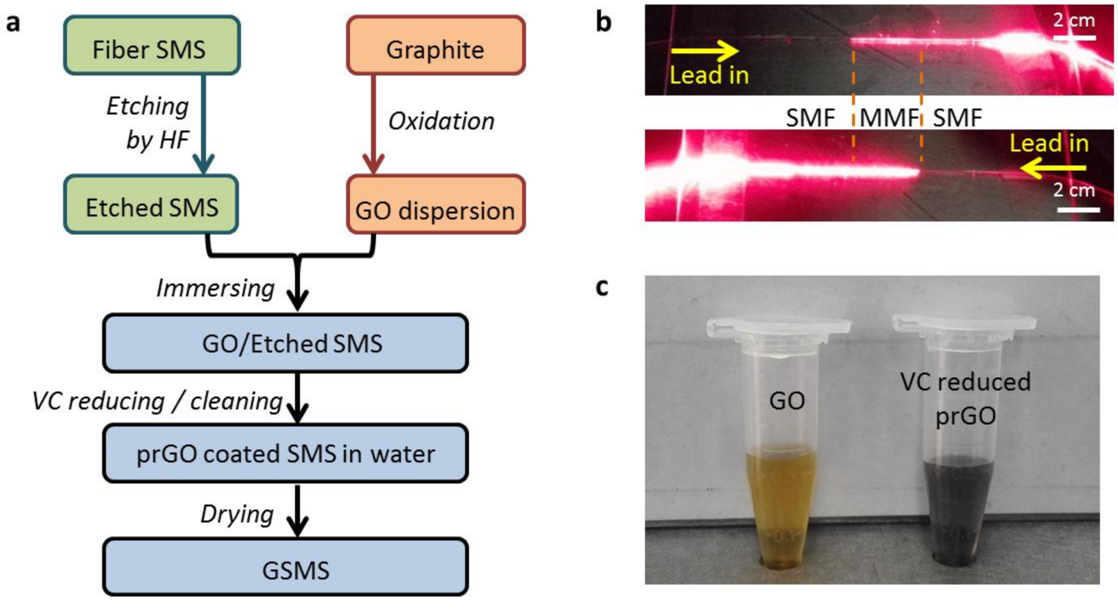

Fig. S2 | Fabrication of the GSMS.

a, Fabrication process. A singlemode-multimode-singlemode structure (SMS) was fabricated by using two section of single mode fiber (SMF-28e, Corning) and a section of multimode fiber (MMF, core diameter 105 µm, Corning), with multimode cavity length of ~3.2 cm. Then, the silica clad of the SMS was etched off by HF, with keeping a 90 µm core.

Firstly, GO was fabricated as following: Graphite powder (2 g) and $NaNO_3$ (1 g) were mixed, then add into concentrated H_2SO_4 (80 mL) with an ice bath. Under vigorous stirring, $KMnO_4$ (8 g) was added slowly to keep the temperature of the mixture below 20℃. After removing the ice bath, the mixture was stirred at 35 ℃ in a water bath for 2 h. Successively, 240 mL of H_2O was slowly added to the pasty and brown mixture. Addition of water into the concentrated H_2SO_4 will release a large amount of heat; therefore, water should be added slowly so that the temperature of the mixture in the ice bath was below 50 ℃. After adding 240 mL of H_2O, 5 mL of 30% H_2O_2 was added to the mixture, then the diluted mixture color changed to brilliant yellow. After continuously stirring for 2 h, the mixture was filtered and washed with 10% HCl aqueous solution(250 mL), DI water, and ethanol (anhydrous) to remove other ions. Finally, the resulting solid was dried by vacuum. Then, the dried GO was dissolved in 40mL DI water with sonication for 2 h to form a uniform dispersion. Then the etched SMS was immersed in the GO dispersion, on a substrate. The water of the GO dispersion was evaporated

naturally in air at room temperature after 24h therefore the thin GO film coated on the fiber was formed. Then the fiber coated by the GO thin film were immersed in 100 mL VC aqueous solution (30g/L), which was heated in a water bath to 80 ^{o}C. After reduced by the hot VC solution for 20 min, the fiber was washed by DI water for several times, and finally dried on a hotplate at 50°C.

b, By using a 633 nm laser, it is obvious that the light energy transmits from the core of the SMF to the surface of the etched MMF. During this process, multimode interferences occur. By launching the 633 nm laser from left side and right side of the SMS respectively, the evanescent light transmitting out of the fiber is obvious, which makes it sensitive to local environment. By using this method, the length of the MMF section could also be conveniently characterized. **c,** Picture of the GO dispersion (yellow) and the partially reduced GO (prGO) dispersion reduced by VC (black).

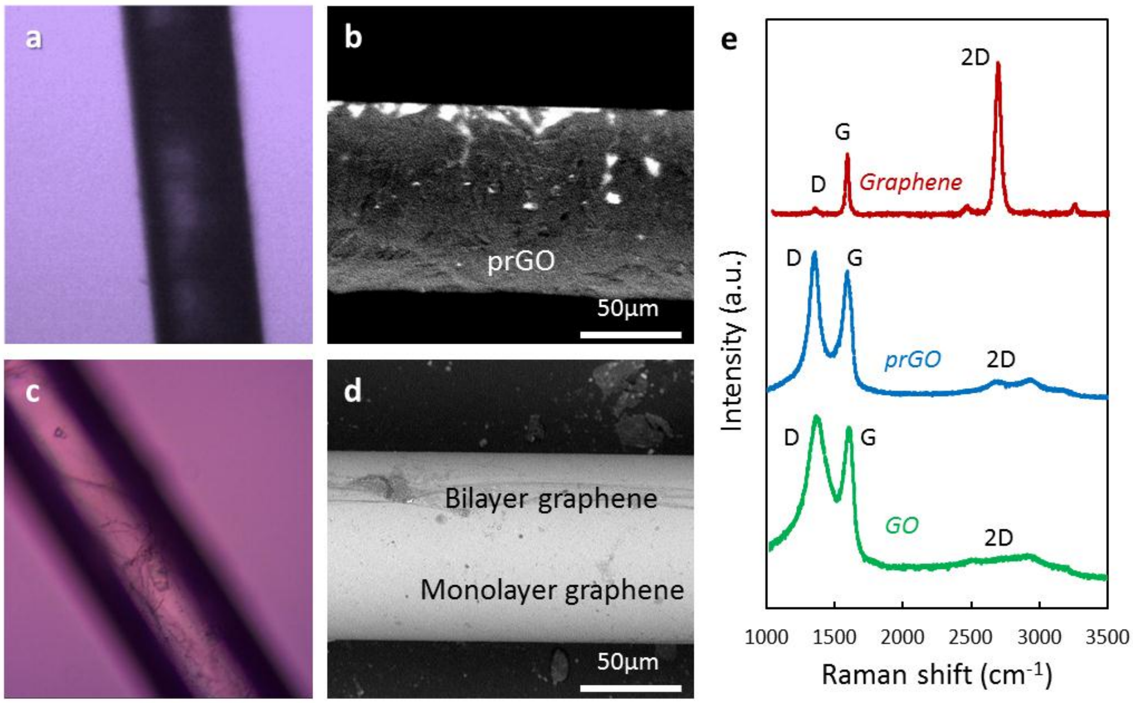

Fig. S3 | Extended characterization: fiber coated by prGO and fiber coated by CVD monolayer graphene.

a & b, Optical microscopy and SEM image of the SMS coated by prGO. Dark prGO is uniformly covered on the surface of the fiber. **c & d,** Optical microscopy and SEM image of the SMS coated by CVD graphene. It is obvious that the CVD graphene is much more

transparent and thinner than the prGO. During the wrapping process, parts of the monolayer graphene are overlapped to be bilayer. **e,** Raman spectra of the prGO (blue curve), GO (green curve) and the CVD graphene (red curve). Both the prGO and graphene demonstrated here are of high quality and uniform on the fiber.

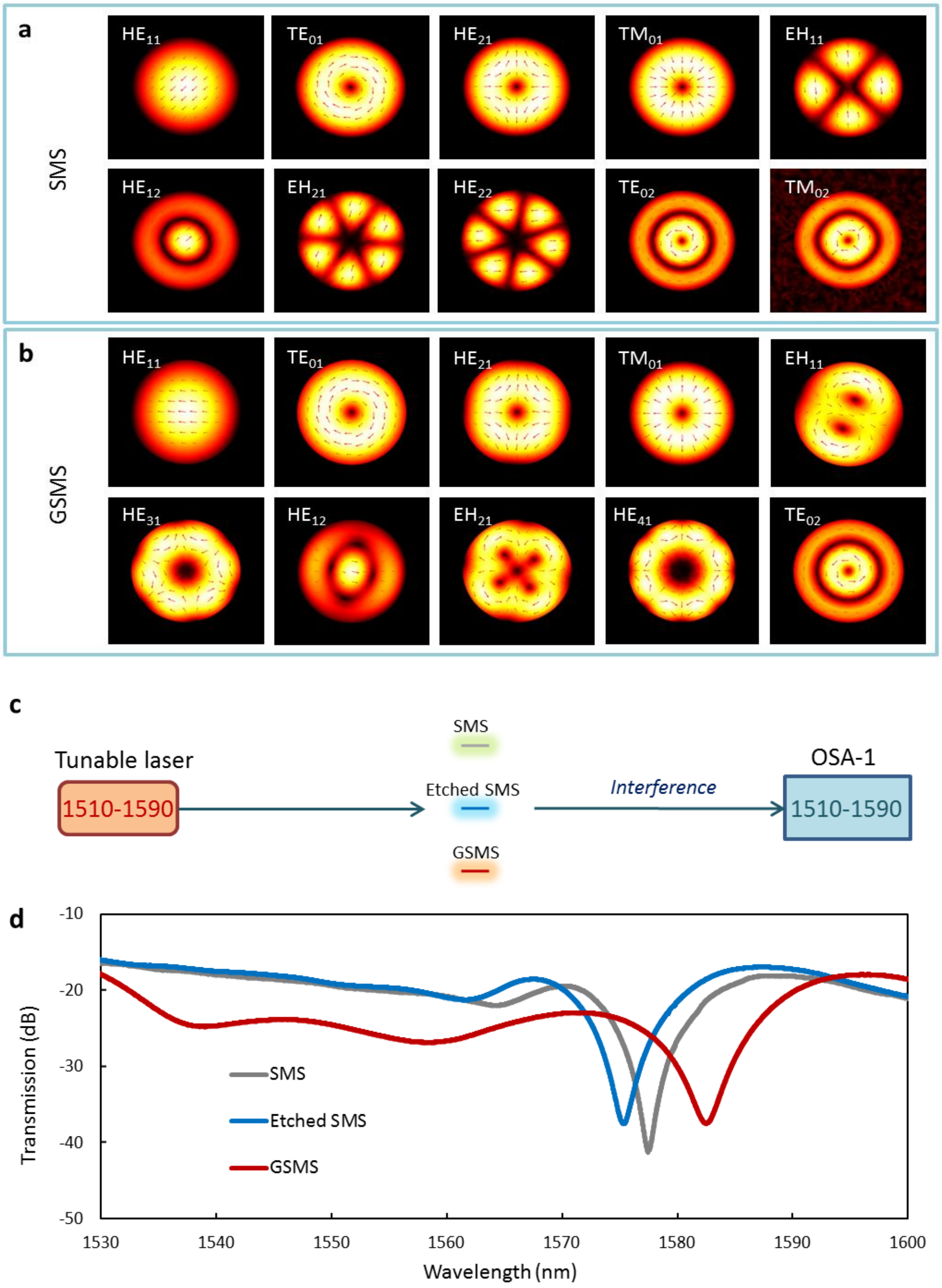

Fig. S4 | Mode distributions and multimode interference.

a, 1st order to 10th order modes of the etched multimode fiber core of SMS, with diameter of 100μm, core index 1.46 and outside index 1.33. **b,** 1st order to 10th order modes of the GSMS, with core diameter of 100 μm and rGO clad thickness of 2 μm. Because of the high index prGO cladding, high order modes in the GSMS are dramatically enhanced. **c&d,** By fixing the length of multimode cavity to be ~3.2 cm, the grey curve, blue curve and red curve shows the transmission spectrum of a typical SMS, etched SMS, and GSMS. Due to the graphene based mode redistribution, the dip of the GSMS shifts and broadens. (**c,** schematic diagram of the measurement system, **d,** measured spectra)

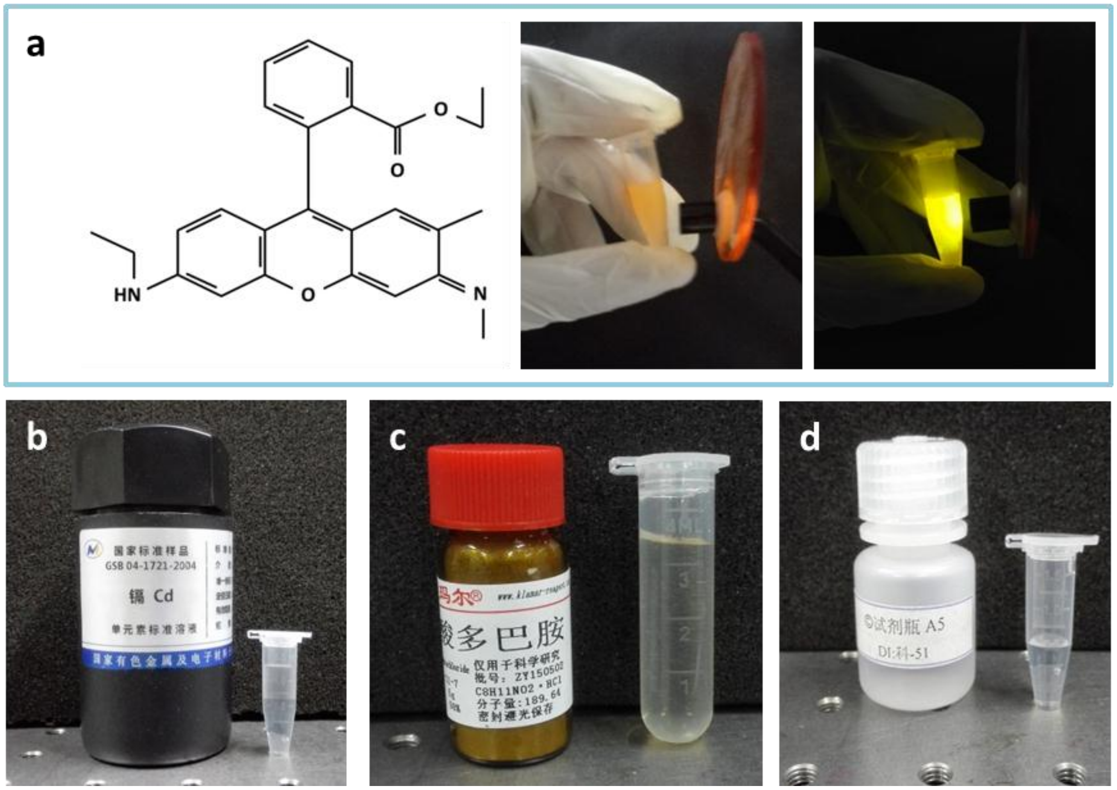

Fig. S5 | Biochemical materials.

a, Rh6G: The Rh6G powder was purchased from J&K Chemical Co. Ltd. (Shanghai), and utilized as the fluorescent donor in the FRET experiment. By using deionized water, we prepared Rh6G aqueous solution with concentration of 1mM. As the photographs show, when exposed in UV light, the Rh6G emitted yellow fluorescence. **b,** Cd^{2+} solution was purchased from Huabiao Standard Reagent Co. Ltd. (Beijing). Considering some specific anion (such as I^- and PO_4^{3-}) might quench the fluorescence of Rh6G, in our

experiment, the Cd^{2+} was in nitrate solutions ($Cd(NO_3)_2$). In this experiment, the Cd^{2+} standard solution (1mg/mL) were diluted with concentration in range of 20 μM to 1 mM. **c,** Dopamine. The dopamine hydrochloride (DA, ASL-279) was purchased from Guide Chem. Ltd., (Shanghai, China). In experiment, the dopamine was dissolved in distilled water. **d,** ssDNA: The Primer synthetized ssDNA (5'-CTC GAC CGA ATT CGT GGC TCT CAT GAC GGA GA-3') solution was purchased from Sangon Biotech. Co. Inc., (Shanghai, China). The ssDNA was dissolved in DI water and with concentration of 200 nM.

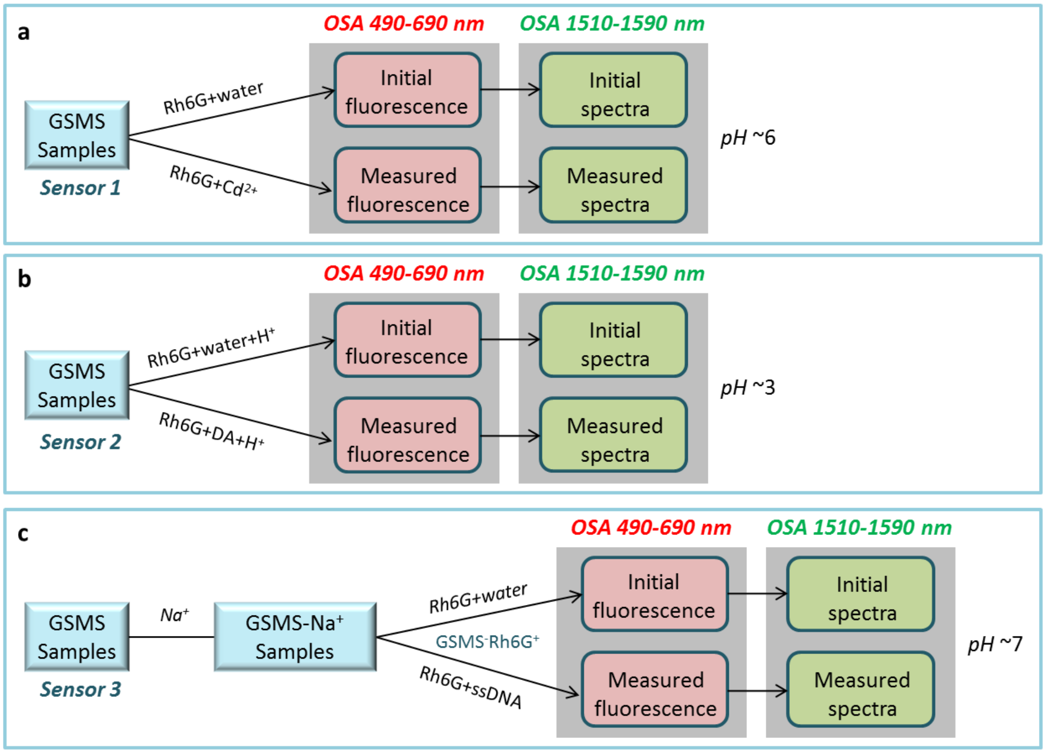

Fig. S6 | Functionalization & measurement.

a, Using *Sensor 1* samples to detect Cd^{2+}. The GSMS samples are measured first by immersing it in Rh6G. Then, Cd^{2+} with different concentration is added in, afterwards the fluorescence and spectra are measured. **b,** Using *Sensor 2* samples to detect DA. The buffer and the GSMS samples are kept pH ~3 by using HNO_3 during the measurement. In the buffer containing H^+, the GSMS samples are measured first by immersing it in Rh6G. Then, DA with different concentration is added in, afterwards the fluorescence and spectra are measured. **c,** Using *Sensor 3* samples to detect ssDNA. Firstly, the GSMS

samples are immersed in 5% Na_2CO_3 solution for 10 min. Then they are cleaned by using enough distilled water to remove excess Na^+. The samples are measured first by immersing it in Rh6G. Then, Cd^{2+} with different concentration is added in, afterwards the fluorescence and spectra are measured.

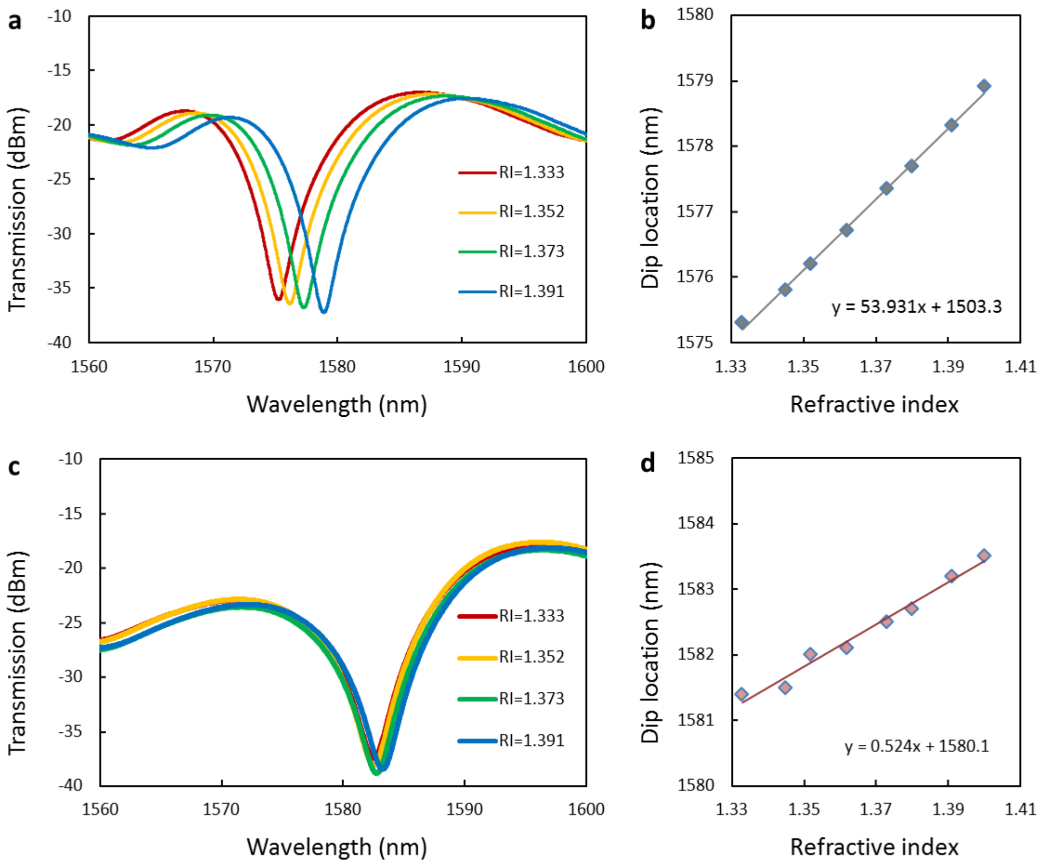

Fig. S7 | Refractive index sensitivity.

a & b, Refractive index sensitivity of an etched SMS sample. By adjusting the refractive index of the solution in the microfluidic channel, the index sensitivity of the SMS is measured. Determined by the evanescent field transmission, the etched SMS shows a sensitivity of ~54 nm/RIU for refractive index detecting, with detection limitation of $\sim 2\times 10^{-5}$ RIU. **c & d,** Refractive index sensitivity of the GSMS. Because of the prGO coating, the evanescent field is tightly contracted, so that it is not sensitive to liquid index (without molecular adsorption). The index sensitivity of the GSMS is ~ 0.5 nm/RIU.

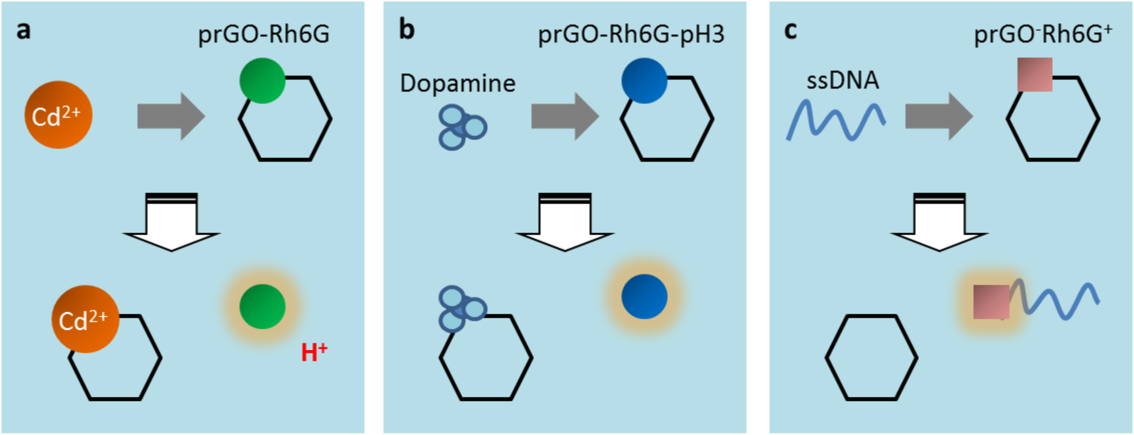

d

$$(Rh6G = NH) + (HOOC - prGO) \rightarrow (Rh6G = N - OC - prGO) + H_2O$$

$$Cd^{2+} + 2(Rh6G = N - OC - prGO) + 2H_2O \rightarrow (prGO - COO^-)_2Cd^{2+} + 2(Rh6G = NH) + 2H^+$$

e

$$(Rh6G = NH) + (HOOC - prGO) \rightarrow (Rh6G = N - OC - prGO) + H_2O$$

$$(DA - OH) + (Rh6G = N - OC - prGO) \rightarrow (DA - OOC - prGO) + (Rh6G = NH)$$

f

$$prGO - COOH + Na^+ \rightarrow (prGO - COO^-)Na^+ + H^+$$

$$(Rh6G = NH) + (prGO - COO^-)Na^+ \rightarrow (prGO - COO^-)(Rh6G - NH^+) + Na^+$$

$$(prGO - COO^-)(Rh6G - NH^+) + (NH_2 - DNA) \rightarrow (prGO - COOH) + (DNA - NH - NH - Rh6G)$$

Fig. S8 | Adsorption and Chemical principles.

a, b & c, Schematic diagram of binding competition between Rh6G and Cd^{2+}, dopamine (DA), ssDNA, respectively. **d, e, f,** Chemical equations for the detection of Cd^{2+}, dopamine (DA), ssDNA, respectively. Specifically, in **d**, Rh6G binds on the prGO first, via –CON= key, as result the fluorescence of the Rh6G is quenched. When Cd^{2+} added in, the binding of the Rh6G and the prGO is freed and the fluorescence of the Rh6G restores, with producing H^+. In **e**, similar to **d**, Rh6G binds on the prGO first, when DA added in, binding competition occurring, Rh6G-prGO turns to be DA-prGO and Rh6G, with fluorescent recovery. In **f**, the prGO is functionalized by Na^+ first, the Rh6G binds the functionalized prGO-Na^+ via ionic bond, with fluorescent quenching. However, the binding of the Rh6G and DNA would be much stronger on the prGO, so that the Rh6G will be took away from the prGO by DNA, with restoring the fluorescence.

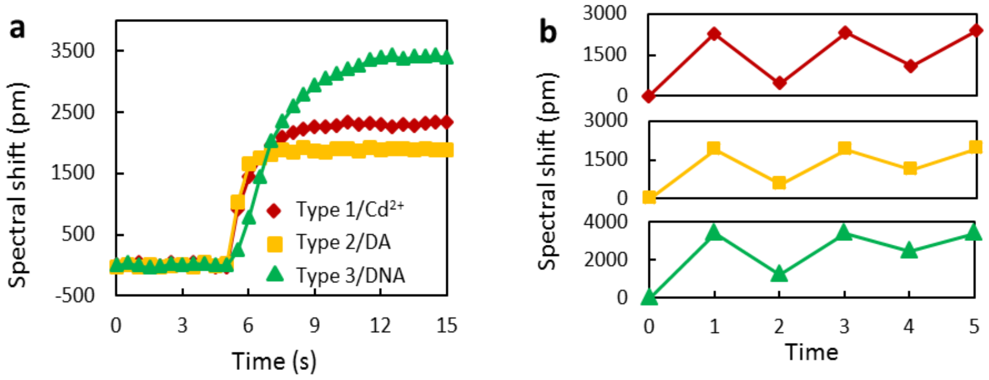

Fig. S9 | Response time and repeatability

a, response time for *Type 1* to detect Cd^{2+} (red diamonds), *Type 2* to detect DA (yellow cubes), *Type 3* to detect ssDNA (green triangles). **b,** shows recoverability of *Type 1* (red diamonds), *Type 2* (yellow cubes), and *Type 3* (green triangles). Dip location dynamics: In Rh6G + DI water (0), in Rh6G + analytes (1), washed by water (2), in Rh6G + analytes again (3), washed by water again (4), in Rh6G + analytes again (5).
